# QSPR analysis of the drugs used to treat major depressive disorder using degree and neighborhood degree based indices

**DOI:** 10.3389/fchem.2026.1784285

**Published:** 2026-05-19

**Authors:** J. J. Jeni Godlin, S. Radha

**Affiliations:** School of Advanced Sciences, Vellore Institute of Technology, Chennai, India

**Keywords:** degree-based indices, edge partitions, major depressive disorder drugs, neighborhood degree-based indices, QSPR, regression analysis

## Abstract

**Introduction:**

The high risk of suicide among major depressive disorder (MDD) patients, together with its substantial contribution to the global burden of disease, emphasizes the necessity of effective drug evaluation strategies. However, traditional clinical trials for MDD drugs are costly and time-consuming, emphasizing the importance of computational approaches such as QSPR modeling for predicting drug behavior and physicochemical properties.

**Methods:**

This study employs degree- and neighborhood degree-based topological indices to characterize the molecular structures of MDD drugs. Topological indices were calculated and analyzed against the physicochemical properties of the drugs using linear, quadratic, and logarithmic regression models to identify the most suitable predictive models, followed by validation to assess their robustness and predictive reliability.

**Results:**

Degree- and neighborhood degree-based topological indices exhibited strong correlations with the physicochemical properties of MDD drugs. Among the linear, quadratic, and logarithmic regression analyses performed, the most suitable predictive models were identified, and model validation confirmed their robustness and predictive reliability.

**Discussion:**

The strong correlations observed between degree- and neighborhood degree-based topological indices and the physicochemical properties of MDD drugs demonstrate the effectiveness of graph-theoretical descriptors in QSPR modeling. The validated predictive models demonstrate the potential of computational approaches to support efficient drug evaluation and rational drug design, reducing reliance on costly and time-consuming experimental studies.

## 1 Introduction

Depression is among the most prevalent mental health disorders and encompasses multiple clinical subtypes with varying degrees of severity; its onset and progression are influenced by a complex interplay of genetic predisposition, individual psychosocial factors, and exposure to stressful or adverse life events. Depression manifests in several distinct clinical forms, including major depressive disorder, persistent depressive disorder, seasonal affective disorder, postpartum depressive disorder, premenstrual dysphoric disorder, and bipolar disorder, each characterized by unique features and clinical trajectories (Cologne and Germany, 2026). Our study focuses on major depressive disorder (MDD), an extreme type of depression. Anhedonia, or a diminished interest in enjoyable activities; feelings of guilt or worthlessness; lack of energy; poor concentration; changes in appetite; psychomotor retardation or agitation; sleep disturbances; or suicidal thoughts are all indicators of this condition. It is a leading global cause of disability, characterized by profound functional impairment and significant deterioration in interpersonal relationships, ultimately diminishing quality of life. MDD was identified by the World Health Organization in 2008 as the third leading contributor to the global burden of disease, and it is projected to surpass all other causes by 2030 (World Health Organization, 2004). MDD patients are more likely to have co-occurring anxiety and drug use problems, which increases their risk of suicide. In addition, it can exacerbate health conditions such as diabetes, hypertension, coronary artery disease, and chronic obstructive pulmonary disease, while also predisposing affected individuals to self-destructive coping behaviors. If left untreated, MDD is often severely debilitating (Bains and Abdijadid, 2023). Therefore, the identification of effective MDD drugs is of significant importance. In this context, QSPR modeling serves as an efficient means for estimating the physicochemical characteristics of potential therapeutic agents, thereby supporting rational drug design.

A few pharmacological agents are used for the treatment of MDD, including trazodone, bupropion, vortioxetine, sertraline, desvenlafaxine, citalopram, fluoxetine, tranylcypromine, mirtazapine, venlafaxine, vilazodone, escitalopram, paroxetine, levomilnacipran (Siwek et al., 2025), aripiprazole (Nelson et al., 2014), cariprazine (Masand et al., 2025), dextromethorphan (McCarthy et al., 2023), olanzapine (El Hussein and Bal, 2025), brexpiprazole (McKeage, 2016), gepirone (Gałka et al., 2025), and maprotiline (Pinder et al., 1977). These agents exert their antidepressant effects through diverse mechanisms, thereby targeting the complex and heterogeneous neurobiological pathways underlying MDD.

Quantitative structure-property relationship (QSPR) is a rigorous computational framework that develops and validates predictive models linking molecular topology to physicochemical properties or biological activity (Katritzky et al., 1995). Once a reliable relationship between molecular structure and activity or property has been established, a large number of compounds, including those not yet synthesized, can be efficiently screened *in silico* to identify candidates with desirable characteristics. This enables the selection of the most promising molecules for synthesis and experimental validation. Consequently, the QSPR approach significantly reduces time and resource expenditure while accelerating the development of new molecules for applications such as pharmaceuticals, materials, and additives (Karelson et al., 1996).

A QSPR model is formulated as a mathematical relationship that links molecular properties to a set of quantitative parameters, known as descriptors, which may be obtained from computational calculations or experimental measurements. These descriptors are correlated with experimentally determined properties using various chemometric techniques to construct statistically robust and predictive QSPR models. Molecular descriptors represent numerical values that encode specific structural information of a molecule and reflect the relationship between chemical constitution and its physical properties, chemical reactivity, or biological activity. Among these, topological indices are two-dimensional molecular descriptors derived solely from the molecular graph (Roy et al., 2015).

Topological descriptors play a vital role in QSPR models, as they quantitatively encode the structural characteristics of molecules, enabling reliable prediction of their physicochemical properties (Arockiaraj et al., 2025a; Balasubramaniyan et al., 2024; Godlin and Radha, 2026; Kara et al., 2025; Pandeeswari and Sankar, 2025; Xu and Liu, 2025). Derived from molecular graphs, these descriptors offer a concise yet highly informative representation of molecular structure (Hayat and Imran, 2014; Nagesh et al., 2025; Tawhari et al., 2025; Ullah et al., 2023). Topological descriptors fall into several general categories, such as degree-based indices, distancebased indices, and others. Numerous studies employing these indices have significantly contributed to the development of the area of chemical graph theory (Ahmed et al., 2025; Anu et al., 2025; Chen et al., 2025; Khan et al., 2025; Naz et al., 2025; Prabhu et al., 2025; Ravi et al., 2021; Sarkarai and Desikan, 2023; Yogalakshmi and Balamurugan, 2025; Zhang et al., 2024). Degree-based indices and neighborhood degree-based indices are vital because they shed light on molecule connectivity and structure, which are critical for comprehending chemical behavior, persistence, and forecasting a variety of attributes. In recent years, several QSPR analyses employing degree- and neighborhood degree-based indices have produced highly reliable models for predicting the properties of chemical structures, enabling the estimation of properties of novel compounds without experimental evaluation, thereby significantly reducing both cost and time (Arockiaraj et al., 2025b; Peter et al., 2025; Qin et al., 2025; Ravi and Chidambaram, 2025; Yousaf and Shahzadi, 2024). Researchers have also performed QSPR studies on pharmacological agents used in the treatment of depressive disorders in general (Kour and Sankar, 2025a; Kour and Sankar, 2025b), with particular emphasis on drugs prescribed for postpartum depressive disorder (Gayathri and Roy, 2025). Our research presents a QSPR analysis of twenty-one drugs used to treat MDD using degree and neighborhood degree indices.

## 2 Materials and methods

### 2.1 Degree and neighborhood degree-based descriptors

Let G represent the molecular graph of the medication used to treat major depressive disorder. Let the sets of vertices and edges of the graph G be denoted by V(G) and E(G) , respectively. The degree of a vertex $v_{i}$ , represented as $d\left(v_{i}\right)$ , is the number of vertices in a molecular graph G that are exactly one unit distant from that vertex $v_{i}$ . The neighborhood degree of a vertex $v_{i}$ is represented by $nd\left(v_{i}\right)$ , which represents the total of the degrees of all vertices that are adjacent to that vertex $v_{i}$ .

Let $d_{G}\left(l,m\right)=|\left\{v_{i}v_{j}\in E\left(G\right):d\left(v_{i}\right)=l\;and\;d\left(v_{j}\right)=m\right\}|$ and let D be a tuple whose entries correspond to the values $d_{G}\left(l,m\right)$ for every ordered pair (l,m) taken from the set of all possible degree-based edge partitions of G , where l and m denote the degrees of the endpoints of an edge $v_{i}v_{j}$ with l≤m .

Let $nd_{G}\left(l,m\right)=|\left\{v_{i}v_{j}\in E\left(G\right):nd\left(v_{i}\right)=l\;and\;nd\left(v_{j}\right)=m\right\}|$ and let ND be a tuple whose entries correspond to the values $nd_{G}\left(l,m\right)$ for every ordered pair (l,m) taken from the set of all possible neighborhood degree-based edge partitions of G , where l and m denote the neighborhood degrees of the endpoints of an edge $v_{i}v_{j}$ with l≤m .

The index function ξ is used to convert degree and neighborhood degree values into topological indices in the following way:

$$\xi^{d}\left(G\right)=\underset{d_{G}\left(l,m\right)\in D}{\sum }d_{G}\left(l,m\right)\xi \left(l,m\right)$$ (1)

$$\xi^{nd}\left(G\right)=\underset{nd_{G}\left(l,m\right)\in ND}{\sum }nd_{G}\left(l,m\right)\xi \left(l,m\right)$$ (2)

where the following indices are represented by ξ(l,m) .

-
$ABC\left(l,m\right)=\sqrt{\frac{l+m-2}{lm}}$ (Atom bond connectivity (Abubakar et al., 2022; Estrada et al., 1998)).
-
$R\left(l,m\right)=\sqrt{\frac{1}{lm}}$ (Randić (Randic; Mondal et al., 2021a)).
-
$M_{1}\left(l,m\right)=l+m$ (First Zagreb (Gutman and Trinajstić, 1972; Mondal et al., 2021b)).
-
$M_{2}\left(l,m\right)=lm$ (Second Zagreb (Gutman and Trinajstić, 1972; Mondal et al., 2019)).
-
$ReZ_{1}\left(l,m\right)=\frac{l+m}{lm}$ (Redefined first Zagreb (Ranjini et al., 2013; Shanmukha et al., 2021)).
-
$ReZ_{2}\left(l,m\right)=\frac{lm}{l+m}$ (Redefined second Zagreb (Ranjini et al., 2013; Shanmukha et al., 2021)).
-
$SO\left(l,m\right)=\sqrt{l^{2}+m^{2}}\;$ (Sombor (Gutman, 2021; Kulli, 2021)).
-
$SS\left(l,m\right)=\sqrt{\frac{lm}{l+m}}\;$ (Shilpa-Shanmukha (Zhao et al., 2021; Arockiyaraj et al., 2025a)).
-
$BMG\left(l,m\right)=\frac{l+m+lm}{\sqrt{lm}}$ (Bi Zagreb-geometric (Arockiaraj et al., 2023; Arockiaraj et al., 2024)).
-
$GBM\left(l,m\right)=\frac{\sqrt{lm}}{l+m+lm}$ (Geometric-bi Zagreb (Arockiaraj et al., 2023; Arockiaraj et al., 2024)).
-
$GA\left(l,m\right)=\frac{2\sqrt{lm}}{l+m}$ (Geometric-arithmetic (Abubakar et al., 2022; Vukičević and Furtula, 2009)).
-
M2m(l,m)=1lm (Modified second Zagreb (Kulli, 2020; Nikolić et al., 2003)).

The Zagreb index, the earliest topological index, was created by Gutman. Later on, the Randić index was developed to quantify carbon-atom branching in saturated hydrocarbons, and the atom-bond connectivity index was developed to study the heat of heptane and octane synthesis. Numerous topological indices have been created in recent years, such as hybrid indices that are formed from geometric, harmonic, and Zagreb indices. New degree- and neighborhood degree-based indices have also emerged as a result of additional changes and redefinitions of already-existing indices. In order to better understand the molecular structures, indices like Sombor and Shilpa-Shanmukha are also introduced. By establishing a computationally viable means of encoding these structural details, these indices provide effective QSPR models to forecast and explain the physicochemical attributes of the chemical structures. The above-mentioned topological indices have been utilized to develop novel best-fitting models for various chemical structures, thereby demonstrating their effectiveness (Arockiaraj et al., 2025b; Farooq et al., 2022; Kirana et al., 2024; Parveen et al., 2022; Paul et al., 2023; Prisc et al., 2025; Rasheed et al., 2024; Sivaranjini and Radha, 2025; Yousaf and Shahzadi, 2024).

**FIGURE 1:**
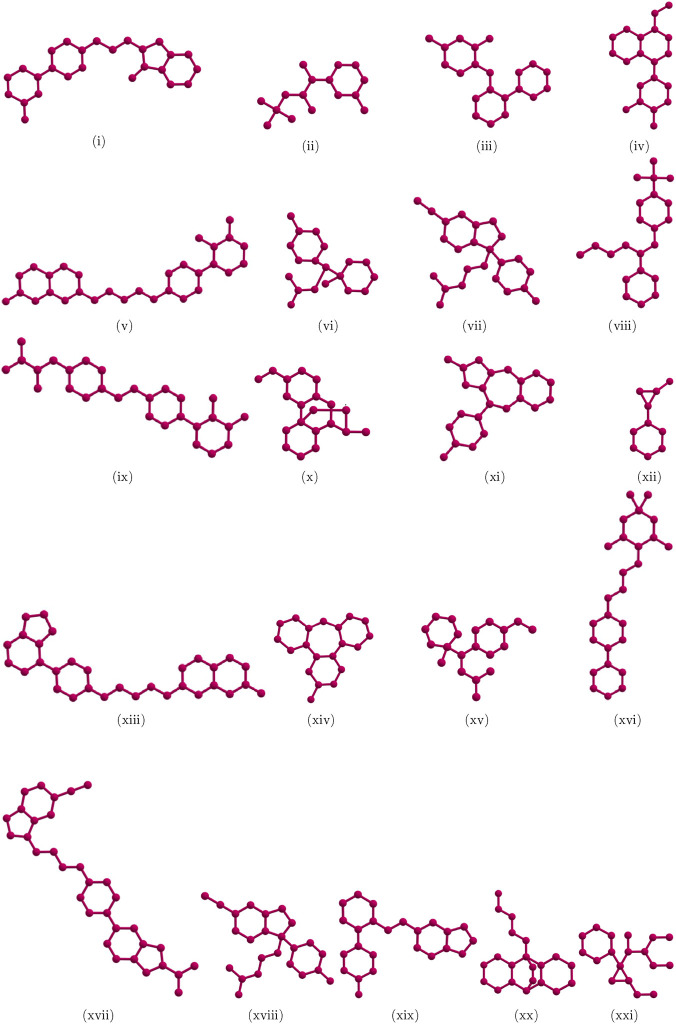
Major depressive disorder drugs: (i) Trazodone (ii) Bupropion (iii) Vortioxetine (iv) Sertraline (v) Aripiprazole (vi) Desvenlafaxine (vii) Citalopram (viii) Fluoxetine (ix) Cariprazine (x) Dextromethorphan (xi) Olanzapine (xii) Tranylcypromine (xiii) Brexpiprazole (xiv) Mirtazapine (xv) Venlafaxine (xvi) Gepirone (xvii) Vilazodone (xviii) Escitalopram (xix) Paroxetine (xx) Maprotiline (xxi) Levomilnacipran.

**FIGURE 2:**
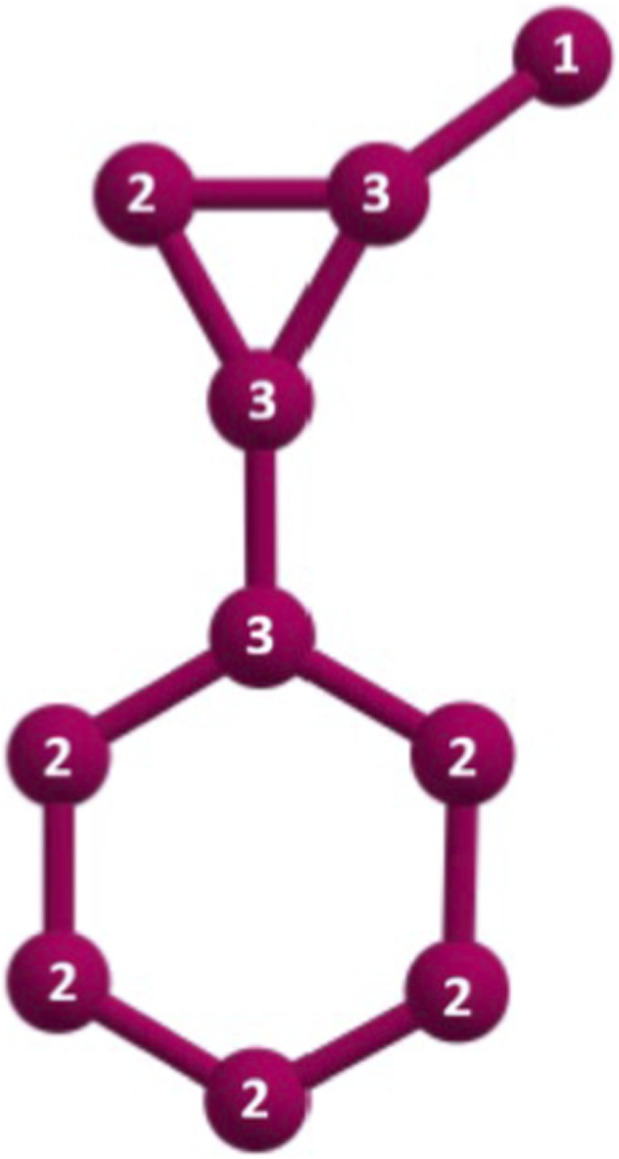
$G_{12}$ with degrees.

In this research, we concentrate on drugs used to treat major depressive disorder, such as trazodone, bupropion, vortioxetine, sertraline, aripiprazole, desvenlafaxine, citalopram, fluoxetine, cariprazine, dextromethorphan, olanzapine, tranylcypromine, brexpiprazole, mirtazapine, venlafaxine, gepirone, vilazodone, escitalopram, paroxetine, maprotiline, and levomilnacipran. Figure 1 shows the molecular graphs of various medications. Using tranylcypromine $\left(G_{12}\right)$ as the reference structure, we now show how the degree-based Randic index $R^{d}$ of $G_{12}$ is computed by edge partitions in Table 1. Figure 2 shows the molecular graph of $G_{12}$ with degrees mentioned in its vertices.

**TABLE 1:** Degree-based partitions of major depressive disorder drug structures.

Graph	Drug	(1,2)	(1,3)	(1,4)	(2,2)	(2,3)	(2,4)	(3,3)	(3,4)
$G_{1}$	Trazodone	-	2	-	9	14	-	4	-
$G_{2}$	Bupropion	-	3	3	2	5	1	2	-
$G_{3}$	Vortioxetine	-	2	-	8	10	-	3	-
$G_{4}$	Sertraline	1	2	-	5	9	-	5	-
$G_{5}$	Aripiprazole	-	3	-	10	16	-	4	-
$G_{6}$	Desvenlafaxine	-	3	1	6	6	2	1	1
$G_{7}$	Citalopram	1	3	-	6	11	2	1	2
$G_{8}$	Fluoxetine	1	-	3	8	9	-	1	1
$G_{9}$	Cariprazine	-	5	-	7	14	-	4	-
$G_{10}$	Dextromethorphan	1	1	-	5	9	2	3	2
$G_{11}$	Olanzapine	-	2	-	5	14	-	4	-
$G_{12}$	Tranylcypromine	-	1	-	4	4	-	2	-
$G_{13}$	Brexpiprazole	-	1	-	12	18	-	4	-
$G_{14}$	Mirtazapine	-	1	-	7	10	-	5	-
$G_{15}$	Venlafaxine	1	2	1	6	7	2	1	1
$G_{16}$	Gepirone	-	2	2	9	10	2	3	-
$G_{17}$	Vilazodone	1	2	-	8	21	-	5	-
$G_{18}$	Escitalopram	1	3	-	6	11	2	1	2
$G_{19}$	Paroxetine	-	1	-	9	14	-	3	-
$G_{20}$	Maprotiline	1	-	-	10	5	2	4	2
$G_{21}$	Levomilnacipran	3	1	-	4	6	1	1	3

In $G_{12}$ , $d_{G}\left(1,3\right)=1$ , $d_{G}\left(2,2\right)=4$ , $d_{G}\left(2,3\right)=4$ , $d_{G}\left(3,3\right)=2$ .

So, $D=\left(d_{G}\left(1,3\right),d_{G}\left(2,2\right),d_{G}\left(2,3\right),d_{G}\left(3,3\right)\right)=\left(1,4,4,2\right)$

$$\begin{array}{ll} R^{d}\left(G_{12}\right) & =\left(1*\sqrt{\frac{1}{1*3}}\right)+\left(4*\sqrt{\frac{1}{2*2}}\right)+\left(4*\sqrt{\frac{1}{2*3}}\right)+\left(2*\sqrt{\frac{1}{3*3}}\right)\\ & =4.88 \end{array}$$

Similarly, all the indices based on the degree mentioned above are evaluated for twenty-one molecular graphs, as presented in Table 4, using Equation 1, degree-based edge partitions in Table 1, and SageMath 9.3 software.

**FIGURE 3:**
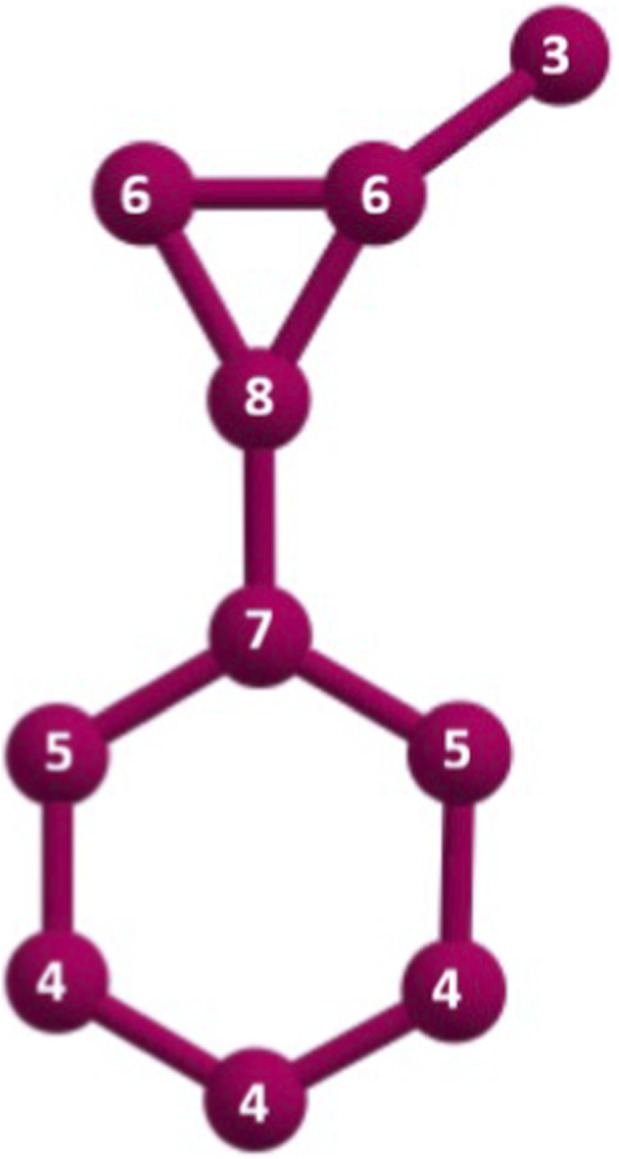
$G_{12}$ with neighborhood degrees.

Figure 3 shows the molecular graph of $G_{12}$ with neighborhood degrees mentioned in its vertices. As with degree-based indices, the neighborhood degree Randic index $R^{nd}$ can be calculated using tranylcypromine $\left(G_{12}\right)$ as the reference structure as $nd_{G}\left(3,6\right)=1$ , $nd_{G}\left(4,4\right)=2$ , $nd_{G}\left(4,5\right)=2$ , $nd_{G}\left(5,7\right)=2$ , $nd_{G}\left(6,6\right)=1$ , $nd_{G}\left(6,8\right)=2$ and $nd_{G}\left(7,8\right)=1$ .

So, $ND=\left(nd_{G}\left(3,6\right),nd_{G}\left(4,4\right),nd_{G}\left(4,5\right),nd_{G}\left(5,7\right),nd_{G}\left(6,6\right),nd_{G}\left(6,8\right),nd_{G}\left(7,8\right)\right)=\left(1,2,2,2,1,2,1\right)$ .

$$\begin{array}{ll} R^{nd}\left(G_{12}\right) & =\left(1*\sqrt{\frac{1}{3*6}}\right)+\left(2*\sqrt{\frac{1}{4*4}}\right)+\left(2*\sqrt{\frac{1}{4*5}}\right)+\left(2*\sqrt{\frac{1}{5*7}}\right)+\left(1*\sqrt{\frac{1}{6*6}}\right)+\left(2*\sqrt{\frac{1}{6*8}}\right)\\ & \;+\left(1*\sqrt{\frac{1}{7*8}}\right)\\ & =2.11 \end{array}$$

Similarly, using Equation 2, neighborhood edge partitions in Tables 2,3 and SageMath 9.3 software, the neighborhood degree-based indices are computed for twenty-one medications and tabulated in Table 5.

**TABLE 2:** Neighborhood degree-based partitions of major depressive disorder drug structures.

Graph	(2,3)	(2,4)	(3,4)	(3,5)	(3,6)	(3,7)	(3,8)	(4,4)	(4,5)	(4,6)	(4,7)	(4,8)	(5,5)	(5,6)	(5,7)
$G_{1}$	-	-	-	1	-	1	-	1	6	-	-	-	3	4	5
$G_{2}$	-	-	-	1	1	1	-	-	5	-	-	-	1	1	2
$G_{3}$	-	-	-	1	1	-	-	3	4	-	-	-	2	1	4
$G_{4}$	-	1	-	-	2	-	-	1	2	-	1	-	2	1	2
$G_{5}$	-	-	-	1	1	1	-	2	4	-	-	-	5	7	4
$G_{6}$	-	-	2	1	-	-	-	2	-	3	-	1	4	-	2
$G_{7}$	-	1	2	1	-	-	-	-	2	2	-	-	5	2	1
$G_{8}$	1	-	1	-	-	-	-	2	3	3	-	-	2	2	3
$G_{9}$	-	-	-	2	2	1	-	-	2	-	-	-	5	10	2
$G_{10}$	-	1	-	-	1	-	-	1	1	2	-	-	1	3	1
$G_{11}$	-	-	-	2	-	-	-	1	2	-	-	-	4	2	4
$G_{12}$	-	-	-	-	1	-	-	2	2	-	-	-	-	-	2
$G_{13}$	-	-	-	1	-	-	-	2	6	-	-	-	5	6	6
$G_{14}$	-	-	-	1	-	-	-	2	4	-	-	-	2	1	2
$G_{15}$	-	1	2	-	-	-	-	2	-	4	-	1	2	2	2
$G_{16}$	-	-	-	-	2	-	-	3	4	2	-	-	2	3	4
$G_{17}$	-	1	-	2	-	-	-	1	2	1	-	-	5	4	9
$G_{18}$	-	1	2	1	-	-	-	-	2	2	-	-	5	2	1
$G_{19}$	-	-	-	1	-	-	-	1	4	​	-	-	6	2	7
$G_{20}$	1	-	1	-	-	-	-	3	4	1	-	-	-	1	-
$G_{21}$	-	3	-	-	-	-	1	2	2	-	2	1	-	-	-

**TABLE 3:** Neighborhood degree-based partitions of major depressive disorder drug structures.

Graph	(5,8)	(5,9)	(6,6)	(6,7)	(6,8)	(6,9)	(6,10)	(7,7)	(7,8)	(7,9)	(7,11)	(8,8)	(8,9)	(8,10)	(8,11)	(9,10)
$G_{1}$	1	-	-	3	-	-	-	2	2	-	-	-	-	-	-	-
$G_{2}$	-	-	-	3	-	-	-	1	-	-	-	-	-	-	-	-
$G_{3}$	1	-	1	3	-	-	-	-	2	-	-	-	-	-	-	-
$G_{4}$	3	-	2	1	-	-	-	-	2	-	-	2	-	-	-	-
$G_{5}$	1	-	1	3	-	-	-	1	2	-	-	-	-	-	-	-
$G_{6}$	-	-	-	-	2	1	-	-	-	1	-	-	1	-	-	-
$G_{7}$	2	1	1	1	-	-	2	-	-	1	-	-	-	1	-	1
$G_{8}$	2	-	1	1	1	-	-	1	-	-	-	-	-	-	-	-
$G_{9}$	1	-	2	1	-	-	-	-	2	​	-	-	-	-	-	-
$G_{10}$	-	1	1	1	2	1	2	-	-	1	-	-	1	-	-	2
$G_{11}$	-	-	-	4	2	-	-	1	2	-	-	1	-	-	-	-
$G_{12}$	-	-	1	-	2	-	-	-	1	-	-	-	-	-	-	-
$G_{13}$	2	-	1	2	-	-	-	1	2	-	-	1	-	-	-	-
$G_{14}$	3	-	-	2	1	-	-	-	2	-	-	3	-	-	-	-
$G_{15}$	-	-	-	-	2	1	-	-	-	1	-	-	1	-	-	-
$G_{16}$	1	-	​	4	2	-	-	1	-	-	-	-	-	-	-	-
$G_{17}$	-	-	1	6	1	-	-	2	2	-	-	-	-	-	-	-
$G_{18}$	2	1	1	1	-	-	2	-	-	1	-	-	-	1	-	1
$G_{19}$	1	-	1	1	-	-	-	1	2	-	-	-	-	-	-	-
$G_{20}$	3	2	-	-	-	-	2	-	-	-	-	2	2	-	-	2
$G_{21}$	2	-	-	-	-	-	-	-	2	-	1	-	-	-	3	-

## 3 Results

### 3.1 QSPR analysis

In this subsection, we do QSPR analysis by correlating the topological indices and physicochemical properties of the major depressive disorder drugs. Table 6 presents the physicochemical properties of the drug compounds, including molecular weight (MW), heavy atom count (HAC), complexity (CO), boiling point (BP), enthalpy of vaporization (EV), flash point (FP), molar refractivity (MR), polarizability (PO), and molar volume (MV), as compiled from the ChemSpider (ChemSpider, 2026) database and PubChem (PubChem, 2026). Among these, molecular weight, heavy atom count, and complexity are computed values generated by PubChem. The remaining properties were predicted using the ACD/Labs Percepta Kernel - PhysChem Module (version 14), as provided by ChemSpider.

#### 3.1.1 Linear models

Here, QSPR analysis is carried out by correlating the index values listed in Tables 4,5 with the physicochemical properties given in Table 6 using linear regression analysis.

**TABLE 4:** Degree-based indices for major depressive disorder drug structures.

Graph	$ABC^{d}$	$R^{d}$	$M_{1}^{d}$	$M_{2}^{d}$	$ReZ_{1}^{d}$	$ReZ_{2}^{d}$	$SO^{d}$	$SS^{d}$	$BMG^{d}$	$GBM^{d}$	$GA^{d}$	^*m*^ $M_{2}^{d}$
$G_{1}$	20.56	12.7	138	162	26	33.3	99.23	30.97	126.95	6.66	28.45	5.69
$G_{2}$	12.04	7.29	78	85	16	16.98	58.5	16.36	71.03	3.62	14.84	3.43
$G_{3}$	16.36	10.24	108	125	21	26	77.74	24.36	99.99	5.32	22.53	4.67
$G_{4}$	15.57	9.7	106	127	20	25.47	76.37	23.53	97.04	5.03	21.49	4.47
$G_{5}$	23.5	14.6	156	181	30	37.45	112.43	35.02	143.98	7.61	32.27	6.61
$G_{6}$	14.53	9.01	96	110	19	22.13	70.4	20.91	87.95	4.59	19.15	4.19
$G_{7}$	18.55	11.55	126	150	24	29.71	91.54	27.62	114.93	5.94	25.18	5.36
$G_{8}$	16.64	10.5	108	121	22	25.08	78.93	23.89	99.94	5.34	22.15	4.94
$G_{9}$	21.6	13.44	142	163	28	33.55	103.06	31.57	131.08	6.9	29.05	6.19
$G_{10}$	16.13	9.74	116	146	20	27.81	83.66	25.14	103.86	5.15	22.49	4.33
$G_{11}$	17.73	10.7	122	146	22	29.3	87.91	26.97	110.95	5.66	24.45	4.69
$G_{12}$	7.81	4.88	52	61	10	12.55	37.38	11.7	48	2.54	10.79	2.22
$G_{13}$	24.7	15.26	166	195	31	40.35	118.97	37.48	152.87	8.06	34.5	6.78
$G_{14}$	16.17	9.83	112	136	20	27.25	80.23	24.94	101.95	5.22	22.66	4.31
$G_{15}$	15.12	9.55	100	115	20	23.25	73.08	21.95	91.94	4.85	20.21	4.53
$G_{16}$	20.21	12.44	134	153	26	31.27	97.75	29.46	122.89	6.42	27.02	5.67
$G_{17}$	26.18	16.1	178	211	33	42.87	128.12	39.68	162.92	8.45	36.25	7.22
$G_{18}$	18.55	11.55	126	150	24	29.71	91.54	27.62	114.93	5.94	25.18	5.36
$G_{19}$	19.08	11.79	128	150	24	31.05	91.82	28.88	117.91	6.21	26.58	5.25
$G_{20}$	16.69	10.37	118	148	21	28.76	84.46	26.12	106.86	5.46	23.71	4.69
$G_{21}$	13.32	8.7	92	114	18	21.93	66.53	20.2	84	4.38	18.49	4.32

**TABLE 5:** Neighborhood degree-based indices for major depressive disorder drug structures.

Graph	$ABC^{nd}$	$R^{nd}$	$M_{1}^{nd}$	$M_{2}^{nd}$	$ReZ_{1}^{nd}$	$ReZ_{2}^{nd}$	$SO^{nd}$	$SS^{nd}$	$BMG^{nd}$	$GBM^{nd}$	$GA^{nd}$	^*m*^ $M_{2}^{nd}$
$G_{1}$	15.99	5.42	324	918	10.95	79.54	231.14	47.82	219.09	3.91	28.72	1.05
$G_{2}$	9.04	3.16	170	454	6.43	41.34	121.81	25.6	116.32	2.24	15.76	0.64
$G_{3}$	12.83	4.45	250	695	8.99	61.39	178.32	37.38	170.32	3.17	22.79	0.9
$G_{4}$	12.1	4.11	254	752	8.38	61.81	181.95	36.59	169.99	2.93	21.66	0.83
$G_{5}$	18.31	6.26	362	1,004	12.66	88.86	258.26	53.93	246	4.5	32.68	1.22
$G_{6}$	11.19	3.93	220	630	7.96	53.63	157.47	32.48	149.13	2.78	19.75	0.82
$G_{7}$	14.35	4.97	300	911	10.09	72.97	214.96	43.11	200.69	3.52	25.64	1.03
$G_{8}$	13	4.68	242	652	9.45	59.3	172.8	36.7	166.25	3.27	22.77	1.03
$G_{9}$	16.74	5.73	326	888	11.63	79.67	233.05	48.71	221.93	4.11	29.62	1.12
$G_{10}$	12.24	4	292	968	8.14	71	209.26	40.01	190.69	2.9	22.65	0.76
$G_{11}$	13.53	4.5	292	875	9.06	72.06	207.79	42.22	195.42	3.27	24.82	0.85
$G_{12}$	6.11	2.11	122	348	4.27	29.87	87.15	18.01	82.61	1.5	10.88	0.43
$G_{13}$	19.25	6.54	390	1,107	13.16	96.15	277.66	57.77	264.14	4.72	34.76	1.26
$G_{14}$	12.44	4.14	272	833	8.36	66.91	193.86	38.96	181.29	3.01	22.81	0.79
$G_{15}$	11.81	4.2	230	657	8.53	55.95	164.8	33.96	156.05	2.94	20.7	0.91
$G_{16}$	15.58	5.35	306	845	10.84	74.89	218.62	45.6	208.01	3.83	27.69	1.06
$G_{17}$	20.23	6.81	422	1,224	13.78	103.87	300.69	61.69	284.02	4.92	36.67	1.32
$G_{18}$	14.35	4.97	300	911	10.09	72.97	214.96	43.11	200.69	3.52	25.64	1.03
$G_{19}$	14.86	5.04	300	845	10.16	73.91	213.66	44.51	203.3	3.64	26.8	0.97
$G_{20}$	12.97	4.46	296	982	9.03	72.03	212.04	41	194.65	3.15	23.69	0.95
$G_{21}$	10.62	3.78	228	741	7.84	54.18	165.12	31.47	150.26	2.59	18.45	0.88

**TABLE 6:** Physicochemical properties of major depressive disorder drugs.

Graph	Drug	MW	HAC	CO	BP ( ° C)	EV	FP	MR	PO	MV
​	​	(g/mol)	​	​	At 760 mmHg	(kJ/mol)	( ° C)	( ${\text{cm}}^{3}$ )	( ${\text{cm}}^{3}$ )	( ${\text{cm}}^{3}$ )
$G_{1}$	Trazodone	371.9	26	611	528.5	80.3	273.4	104.2	41.3	278.8
$G_{2}$	Bupropion	239.74	16	247	334.8	57.8	156.3	67.9	26.9	224.7
$G_{3}$	Vortioxetine	298.4	21	316	424.8	67.9	210.7	92.7	36.8	256.5
$G_{4}$	Sertraline	306.2	20	322	416.3	67	205.6	85.8	34	243.9
$G_{5}$	Aripiprazole	448.4	30	559	646.2	95.3	344.6	120.3	47.7	355
$G_{6}$	Desvenlafaxine	263.37	19	266	403.8	69.1	193.2	77.8	30.9	236.1
$G_{7}$	Citalopram	324.4	24	466	428.3	68.3	212.8	92.1	36.5	272.6
$G_{8}$	Fluoxetine	309.33	22	308	395.1	64.5	192.8	79.9	31.7	266.7
$G_{9}$	Cariprazine	427.4	28	491	600.1	89.3	316.7	117.2	46.4	344.5
$G_{10}$	Dextromethorphan	271.4	20	370	394.9	64.5	116.2	81.8	32.4	243.8
$G_{11}$	Olanzapine	312.4	22	432	476	74	241.7	92.2	36.5	236
$G_{12}$	Tranylcypromine	133.19	10	116	218.3	45.5	90.8	41.8	16.6	125
$G_{13}$	Brexpiprazole	433.6	31	636	675.2	99.1	362.1	126.3	50.1	348
$G_{14}$	Mirtazapine	265.35	20	345	432.4	68.8	215.3	80.7	32	216.6
$G_{15}$	Venlafaxine	277.4	20	279	397.6	68.3	194.2	82.6	32.8	261.7
$G_{16}$	Gepirone	359.5	26	476	562.3	84.5	293.8	99	39.3	314.7
$G_{17}$	Vilazodone	441.5	33	729	745.1	108.6	404.4	128.7	51	328.8
$G_{18}$	Escitalopram	324.4	24	466	428.3	68.3	212.8	92.1	36.5	272.6
$G_{19}$	Paroxetine	329.4	24	402	451.7	71.1	227	87.9	34.9	271.5
$G_{20}$	Maprotiline	277.4	21	339	399.6	65	187.7	87.8	34.8	256.7
$G_{21}$	Levomilnacipran	246.35	18	295	393	64.3	191.5	73.2	29	228.6

The linear regression model is given by Þ $=a_{1}+a_{2}\xi$ , where Þ denotes the physicochemical attribute, ξ represents the index, and $a_{1}$ and $a_{2}$ denote the regression constants. By fitting and evaluating this model for each index listed in Tables 4,5 against the corresponding physicochemical property values in Table 6, the obtained correlation coefficients are summarized in Tables 7,8.

**TABLE 7:** Linear: correlation coefficient between degree based indices and properties.

Indices	MW	HAC	CO	BP	EV	FP	MR	PO	MV
$ABC^{d}$	0.9725	0.9962	0.9568	0.9609	0.9476	0.9279	0.9767	0.9769	0.9247
$R^{d}$	0.9756	0.9983	0.9511	0.9594	0.9466	0.9313	0.9775	0.9777	0.9338
$M_{1}^{d}$	0.9518	0.9855	0.9612	0.9497	0.9344	0.9042	0.968	0.9681	0.9
$M_{2}^{d}$	0.9139	0.9585	0.9511	0.9218	0.904	0.8633	0.9451	0.9449	0.8581
$ReZ_{1}^{d}$	0.98128	1	0.9479	0.9601	0.948	0.9337	0.97767	0.9779	0.9457
$ReZ_{2}^{d}$	0.9373	0.9742	0.9565	0.9413	0.925	0.8948	0.96	0.96	0.8766
$SO^{d}$	0.9567	0.9889	0.9616	0.9518	0.9369	0.9067	0.9699	0.9699	0.9091
$SS^{d}$	0.9514	0.984	0.9584	0.9503	0.9351	0.9092	0.9678	0.9679	0.8943
$BMG^{d}$	0.9583	0.9892	0.9604	0.9539	0.939	0.9128	0.9715	0.9716	0.9062
$GBM^{d}$	0.967	0.9935	0.9546	0.9576	0.9439	0.9257	0.9746	0.9748	0.9167
$GA^{d}$	0.9561	0.9866	0.9562	0.9527	0.9379	0.9158	0.9698	0.97	0.8998
^*m*^ $M_{2}^{d}$	0.977	0.9959	0.9358	0.9505	0.9388	0.9284	0.9721	0.9723	0.9509

**TABLE 8:** Linear: correlation coefficient between neighborhood degree based indices and properties.

Indices	MW	HAC	CO	BP	EV	FP	MR	PO	MV
$ABC^{nd}$	0.9734	0.9968	0.9534	0.9604	0.9473	0.9312	0.977	0.9773	0.9272
$R^{nd}$	0.9758	0.9976	0.9385	0.9531	0.9416	0.9335	0.9732	0.9736	0.9424
$M_{1}^{nd}$	0.9139	0.9585	0.9511	0.9218	0.904	0.8633	0.9451	0.9449	0.8581
$M_{2}^{nd}$	0.7933	0.8607	0.8828	0.8161	0.7945	0.7328	0.8522	0.8515	0.7447
$ReZ_{1}^{nd}$	0.9764	0.9977	0.9386	0.9533	0.9416	0.933	0.9736	0.974	0.9448
$ReZ_{2}^{nd}$	0.9151	0.9594	0.9521	0.9242	0.9066	0.8671	0.9459	0.9458	0.8558
$SO^{nd}$	0.9131	0.9578	0.9504	0.9204	0.9024	0.8613	0.9444	0.9442	0.859
$SS^{nd}$	0.9451	0.9803	0.9594	0.9462	0.9304	0.9003	0.9645	0.9646	0.888
$BMG^{nd}$	0.9305	0.9708	0.9564	0.9349	0.918	0.8825	0.9561	0.956	0.8748
$GBM^{nd}$	0.9762	0.9977	0.9457	0.9582	0.9462	0.9353	0.9756	0.976	0.9347
$GA^{nd}$	0.9628	0.9912	0.9573	0.9564	0.9423	0.9209	0.9728	0.973	0.9093
^*m*^ $M_{2}^{nd}$	0.9395	0.9667	0.8808	0.8994	0.89	0.892	0.9318	0.9323	0.9407

The best predictive linear regression models with their correlation coefficients are given below:

MW=14.333ReZ1d−7.0127, r=0.98128HAC=ReZ1d, r=1CO=6.9043SOd−191.6, r=0.9616BP=27.514ABCd−21.742, r=0.9609EV=2.6342ReZ1d+13.823, r=0.948FP=89.714GBMnd−70.132, r=0.9353MR=3.7684ReZ1d+5.8105, r=0.97767PO=1.493ReZ1d+2.3298, r=0.9779MV=43.724M2dm+47.344, r=0.9509

#### 3.1.2 Quadratic models

Here, QSPR analysis is carried out by correlating the index values listed in Tables 4,5 with the physicochemical properties given in Table 6 using quadratic regression analysis.

The quadratic regression model is given by Þ $=a_{1}+a_{2}\xi +a_{3}\xi^{2}$ , where Þ denotes the physicochemical attribute, ξ represents the index, and $a_{1}$ , $a_{2}$ , and $a_{3}$ denote the regression constants. By fitting and evaluating this model for each index listed in Tables 4,5 against the corresponding physicochemical property values in Table 6, the obtained correlation coefficients are summarized in Tables 9,10.

**TABLE 9:** Quadratic: correlation coefficient between degree based indices and properties.

Indices	MW	HAC	CO	BP	EV	FP	MR	PO	MV
$ABC^{d}$	0.9727	0.9963	0.9607	0.97239	0.966	0.9459	0.9767	0.9769	0.9301
$R^{d}$	0.9757	0.9983	0.9553	0.971	0.965	0.9489	0.9775	0.9777	0.9386
$M_{1}^{d}$	0.9519	0.9855	0.9668	0.963	0.9554	0.9253	0.9681	0.9681	0.9047
$M_{2}^{d}$	0.914	0.9589	0.9596	0.9381	0.9289	0.8879	0.9456	0.9454	0.8612
$ReZ_{1}^{d}$	0.98131	1	0.9525	0.97236	0.9671	0.9519	0.9777	0.97794	0.9503
$ReZ_{2}^{d}$	0.9373	0.9742	0.9618	0.9547	0.9463	0.916	0.9601	0.9601	0.8804
$SO^{d}$	0.9567	0.9889	0.9676	0.9655	0.9582	0.9283	0.97	0.97	0.9138
$SS^{d}$	0.9515	0.984	0.9629	0.9625	0.9547	0.9287	0.9679	0.968	0.8988
$BMG^{d}$	0.9583	0.9892	0.9652	0.9663	0.9589	0.9327	0.9716	0.9716	0.911
$GBM^{d}$	0.9672	0.9936	0.9583	0.9686	0.9618	0.943	0.9746	0.9748	0.9216
$GA^{d}$	0.9562	0.9866	0.9602	0.9642	0.9567	0.9342	0.9698	0.97	0.9043
^*m*^ $M_{2}^{d}$	0.9771	0.996	0.9422	0.9648	0.9601	0.9485	0.9724	0.9726	0.954

**TABLE 10:** Quadratic: correlation coefficient between neighborhood degree based indices and properties.

Indices	MW	HAC	CO	BP	EV	FP	MR	PO	MV
$ABC^{nd}$	0.9736	0.9969	0.957	0.9713	0.9649	0.9481	0.977	0.9773	0.9323
$R^{nd}$	0.9758	0.9976	0.9435	0.9656	0.9607	0.951	0.9732	0.9736	0.9463
$M_{1}^{nd}$	0.914	0.9589	0.9596	0.9381	0.9289	0.8879	0.9456	0.9454	0.8612
$M_{2}^{nd}$	0.7935	0.861	0.8931	0.8285	0.8146	0.748	0.8525	0.8518	0.7504
$ReZ_{1}^{nd}$	0.9764	0.9977	0.944	0.9662	0.9613	0.9511	0.9737	0.9741	0.9485
$ReZ_{2}^{nd}$	0.9151	0.9596	0.9595	0.9394	0.9304	0.8906	0.9462	0.9461	0.8595
$SO^{nd}$	0.9131	0.9584	0.9594	0.9372	0.928	0.8864	0.9451	0.9448	0.8618
$SS^{nd}$	0.9452	0.9803	0.9646	0.9592	0.9512	0.9211	0.9646	0.9646	0.8924
$BMG^{nd}$	0.9305	0.971	0.9634	0.9498	0.9412	0.9056	0.9564	0.9563	0.8783
$GBM^{nd}$	0.9764	0.9978	0.9494	0.9692	0.9636	0.9514	0.9756	0.976	0.9396
$GA^{nd}$	0.963	0.9912	0.9612	0.9677	0.9607	0.9389	0.9728	0.973	0.9145
^*m*^ $M_{2}^{nd}$	0.9417	0.9694	0.896	0.9224	0.9209	0.9203	0.9346	0.9351	0.9411

The best predictive quadratic regression models with their correlation coefficients are given below:

MW=−0.0147ReZ1d2+14.995ReZ1d−14.051, r=0.98131HAC=ReZ1d, r=1CO=0.025SOd2+2.6514SOd−20.535, r=0.9676BP=0.6958ABCd2+2.9662ABCd+182.64, r=0.97239EV=0.0685ReZ1d2−0.441ReZ1d+46.53, r=0.9671FP=0.3522ReZ1d2−2.0866ReZ1d+88.229, r=0.9519MR=0.0041ReZ1d2+3.5829ReZ1d+7.7834, r=0.9777PO=0.0016ReZ1d2+1.4191ReZ1d+3.1154, r=0.97794MV=−2.0656M2dm2+64.081M2dm−0.204, r=0.954

#### 3.1.3 Logarithmic models

Here, QSPR analysis is carried out by correlating the index values listed in Tables 4,5 with the physicochemical properties given in Table 6 using logarithmic regression analysis.

The logarithmic regression model is given by Þ $=a_{1}+a_{2}ln\left(\xi \right)$ , where Þ denotes the physicochemical attribute, ξ represents the index, and $a_{1}$ and $a_{2}$ denote the regression constants. By fitting and evaluating this model for each index listed in Tables 4,5 against the corresponding physicochemical property values in Table 6, the obtained correlation coefficients are summarized in Tables 11,12.

**TABLE 11:** Logarithmic: correlation coefficient between degree based indices and properties.

Indices	MW	HAC	CO	BP	EV	FP	MR	PO	MV
$ABC^{d}$	0.9556	0.9776	0.9184	0.9146	0.8948	0.8742	0.9587	0.9589	0.9274
$R^{d}$	0.9573	0.979	0.9128	0.9133	0.8941	0.8779	0.9586	0.9588	0.9346
$M_{1}^{d}$	0.9334	0.964	0.9181	0.9008	0.8788	0.8484	0.9473	0.9473	0.9021
$M_{2}^{d}$	0.8953	0.9356	0.905	0.8728	0.8485	0.8092	0.9227	0.9225	0.8597
$ReZ_{1}^{d}$	0.9609	0.9783	0.9077	0.912	0.8936	0.8783	0.957	0.9572	0.945
$ReZ_{2}^{d}$	0.9205	0.9552	0.9157	0.8946	0.8715	0.8412	0.9409	0.9408	0.8789
$SO^{d}$	0.9368	0.9655	0.9168	0.9012	0.8798	0.8493	0.9478	0.9478	0.9101
$SS^{d}$	0.9351	0.9658	0.9191	0.9043	0.8823	0.8558	0.9497	0.9498	0.8971
$BMG^{d}$	0.9406	0.9691	0.9193	0.9062	0.8847	0.858	0.9521	0.9521	0.9085
$GBM^{d}$	0.9509	0.9764	0.9176	0.9129	0.8926	0.8735	0.9575	0.9577	0.9192
$GA^{d}$	0.9403	0.9695	0.9185	0.9077	0.8863	0.8634	0.9525	0.9527	0.9025
^*m*^ $M_{2}^{d}$	0.9534	0.9716	0.8938	0.9012	0.8833	0.872	0.9488	0.949	0.9466

**TABLE 12:** Logarithmic: correlation coefficient between neighborhood degree based indices and properties.

Indices	MW	HAC	CO	BP	EV	FP	MR	PO	MV
$ABC^{nd}$	0.9569	0.9793	0.9165	0.9156	0.896	0.879	0.9598	0.96	0.9296
$R^{nd}$	0.9569	0.9779	0.9005	0.9077	0.89	0.8811	0.9541	0.9545	0.9418
$M_{1}^{nd}$	0.8953	0.9356	0.905	0.8728	0.8485	0.8092	0.9227	0.9225	0.8597
$M_{2}^{nd}$	0.7886	0.8474	0.8422	0.7818	0.7549	0.6995	0.839	0.8384	0.7574
$ReZ_{1}^{nd}$	0.9562	0.9767	0.8996	0.9068	0.889	0.8795	0.9534	0.9537	0.9432
$ReZ_{2}^{nd}$	0.898	0.9382	0.9076	0.8761	0.852	0.8135	0.9251	0.9249	0.8589
$SO^{nd}$	0.8937	0.9342	0.9035	0.871	0.8465	0.8069	0.9213	0.921	0.8598
$SS^{nd}$	0.9282	0.9607	0.9181	0.8989	0.8764	0.8461	0.9452	0.9452	0.8908
$BMG^{nd}$	0.912	0.9488	0.9119	0.886	0.8625	0.8275	0.9345	0.9344	0.8763
$GBM^{nd}$	0.9601	0.9808	0.91	0.9145	0.8962	0.8844	0.959	0.9593	0.9368
$GA^{nd}$	0.9472	0.9739	0.9196	0.9112	0.8905	0.8683	0.9558	0.956	0.9128
^*m*^ $M_{2}^{nd}$	0.9127	0.9377	0.8371	0.8507	0.8358	0.8366	0.9061	0.9065	0.9303

The best predictive logarithmic regression models with their correlation coefficients are given below:

MW=287.12lnReZ1d−569.85, r=0.9609HAC=19.583lnGBMnd−0.4626, r=0.9808CO=497.67lnGAnd−1172.7, r=0.9196BP=420.95lnABCnd−623.97, r=0.9156EV=49.715lnGBMnd+14.806, r=0.8962FP=259.79lnGBMnd−75.563, r=0.8844MR=73.474lnABCnd−98.92, r=0.9598PO=29.11lnABCnd−39.163, r=0.96MV=195.49lnM2dm−43.102, r=0.9466

#### 3.1.4 Comparison of regression models

Among the above-discussed linear, quadratic, and logarithmic models, the linear regression between $ReZ_{1}^{d}$ and HAC yields a correlation coefficient of 1, indicating a perfect linear relationship. Hence, higher-order regression models, including the quadratic model, are not required for this case. A heavy atom count (HAC) represents the total number of non-hydrogen atoms in a molecule. In our study, hydrogen-suppressed molecular graphs are considered for the QSPR analysis. Unlike other indices, $ReZ_{1}^{d}$ mathematically simplifies to a basic atom count for the drugs analyzed. Since hydrogen atoms are excluded, both measures count the same set of atoms, resulting in their equivalence. Quadratic models were more effective for other physicochemical properties, as evidenced by their highest correlation values. The best-performing linear and quadratic models used to predict the physicochemical properties of the drugs are analyzed with IBM SPSS Statistics (Version 27) software, and the resulting r , $r^{2}$ , $adjr^{2}$ , F , and standard error SE values are summarized in Table 13.

- MW is most accurately predicted by the degree-based redefined first Zagreb index $ReZ_{1}^{d}$ .
- HAC is most accurately predicted by the degree-based redefined first Zagreb index $ReZ_{1}^{d}$ .
- CO is most accurately predicted by the degree-based Sombor index $SO^{d}$ .
- BP is most accurately predicted by the degree-based atom bond connectivity index $ABC^{d}$ .
- EV is most accurately predicted by the degree-based redefined first Zagreb index $ReZ_{1}^{d}$ .
- FP is most accurately predicted by the degree-based redefined first Zagreb index $ReZ_{1}^{d}$ .
- MR is most accurately predicted by the degree-based redefined first Zagreb index $ReZ_{1}^{d}$ .
- PO is most accurately predicted by the degree-based redefined first Zagreb index $ReZ_{1}^{d}$ .
- MO is most accurately predicted by the degree-based modified second Zagreb index ^*m*^ $M_{2}^{d}$ .

**TABLE 13:** Best-fitted regression models.

Best fitted models	r	$r^{2}$	$adj\;r^{2}$	F	SE
$MW=-0.0147{\left(ReZ_{1}^{d}\right)}^{2}+14.995\left(ReZ_{1}^{d}\right)-14.051$	0.9813	0.963	0.9589	234.0981	15.6895
$HAC=ReZ_{1}^{d}$	1	1	1	Undefined	0
$CO=0.025{\left(SO^{d}\right)}^{2}+2.6514\left(SO^{d}\right)-20.535$	0.9676	0.9362	0.9291	132.0202	39.2379
$BP=0.6958{\left(ABC^{d}\right)}^{2}+2.9662\left(ABC^{d}\right)+182.64$	0.9724	0.9455	0.9395	156.2866	30.1562
$EV=0.0685{\left(ReZ_{1}^{d}\right)}^{2}-0.441\left(ReZ_{1}^{d}\right)+46.53$	0.9671	0.9352	0.928	129.8767	3.9487
$FP=0.3522{\left(ReZ_{1}^{d}\right)}^{2}-2.0866\left(ReZ_{1}^{d}\right)+88.229$	0.9519	0.9062	0.8957	86.9032	25.1615
$MR=0.0041{\left(ReZ_{1}^{d}\right)}^{2}+3.5829\left(ReZ_{1}^{d}\right)+7.7834$	0.9777	0.9559	0.951	195.1005	4.5184
$PO=0.0016{\left(ReZ_{1}^{d}\right)}^{2}+1.4191\left(ReZ_{1}^{d}\right)+3.1154$	0.9779	0.9564	0.9515	197.2211	1.7805
MV=−2.0656(M2dm)2+64.081(M2dm)−0.204	0.954	0.9101	0.9001	91.07	16.6975

**FIGURE 4:**
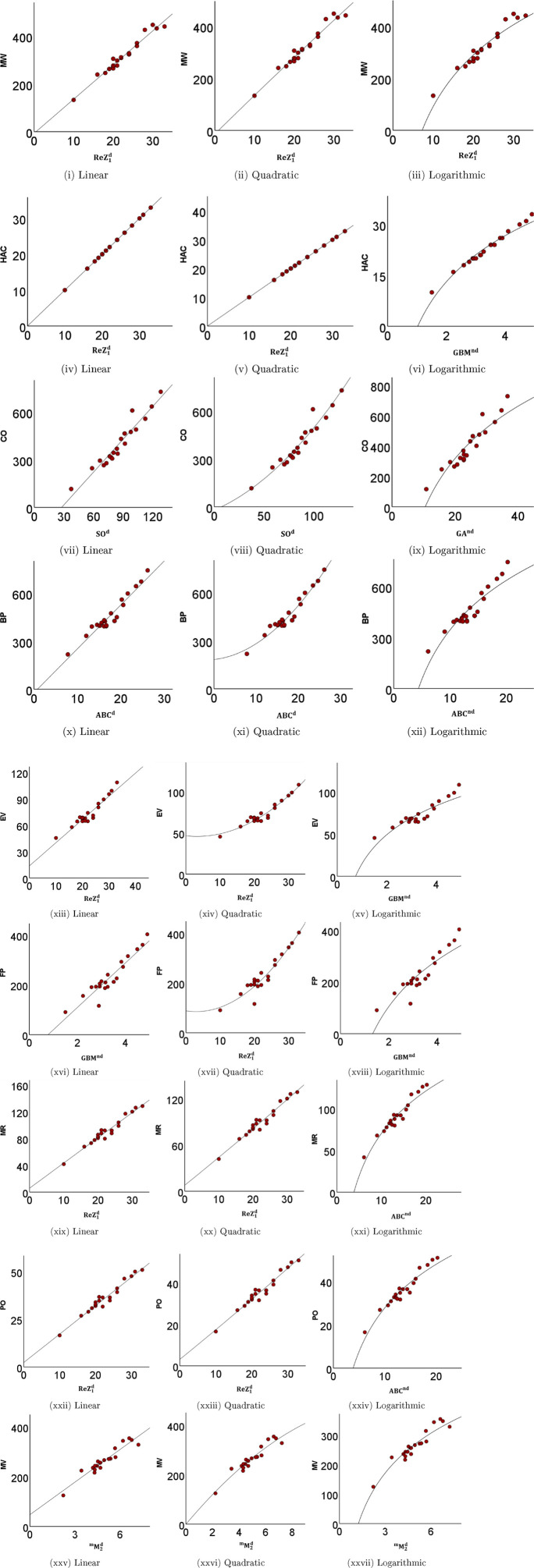
Scatter plots comparing the regression models.

**FIGURE 5:**
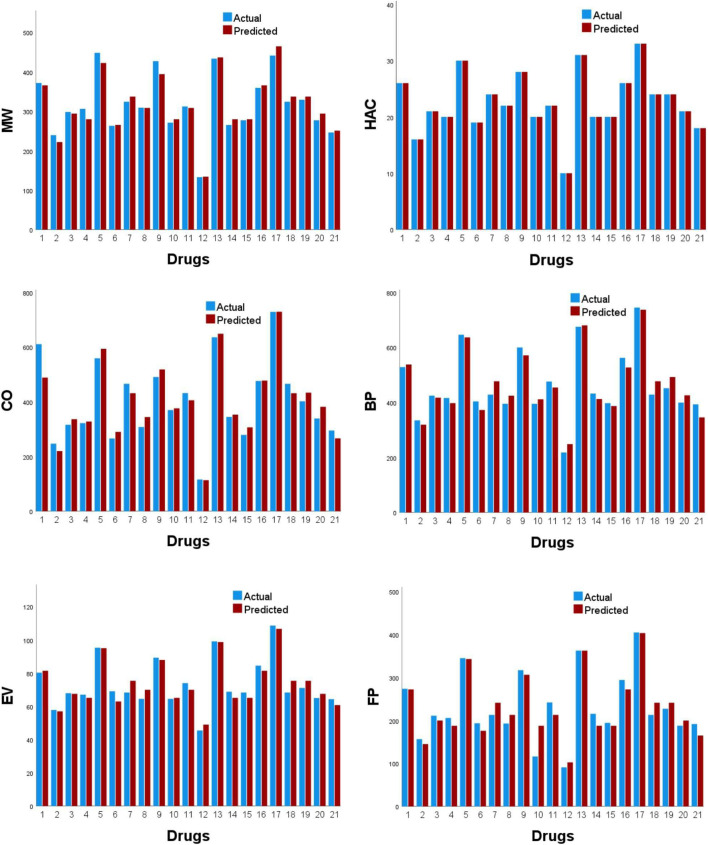
Plots showing the comparison of physicochemical properties.

**FIGURE 6:**
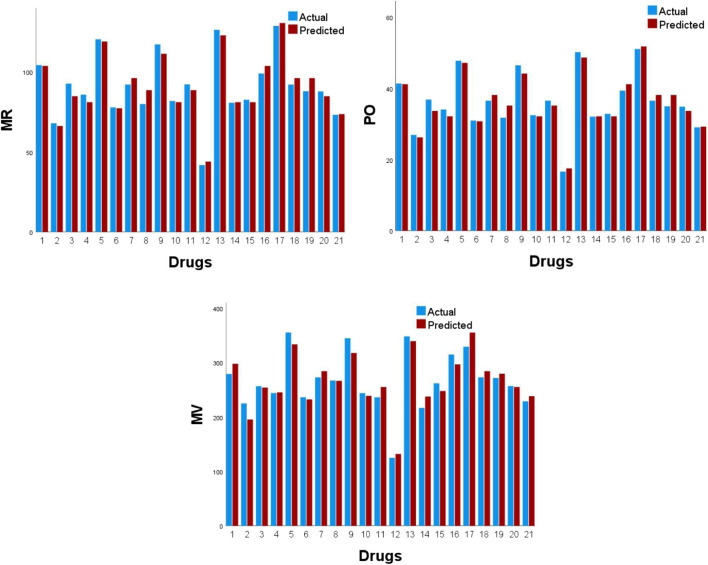
Plots showing the comparison of physicochemical properties.

Figure 4 displays scatter plots of the best predictive linear, quadratic, and logarithmic models of the physicochemical attributes. The experimentally observed physicochemical attributes with the corresponding values predicted by the optimal models are presented in Tables 14,15, while their graphical comparison is illustrated in Figures 5, 6.

**TABLE 14:** Comparison of physico-chemical properties with their predicted values.

Properties	MW		HAC		CO		BP	
Drug	Actual	Predicted	Actual	Predicted	Actual	Predicted	Actual	Predicted
Trazodone	371.9	365.9	26	26	611	488.7	528.5	537.8
Bupropion	239.7	222.1	16	16	247	220.1	334.8	319.2
Vortioxetine	298.4	294.4	21	21	316	336.6	424.8	417.4
Sertraline	306.2	280	20	20	322	327.7	416.3	397.6
Aripiprazole	448.4	422.6	30	30	559	593.6	646.2	636.6
Desvenlafaxine	263.4	265.5	19	19	266	290	403.8	372.6
Citalopram	324.4	337.4	24	24	466	431.7	428.3	477.1
Fluoxetine	309.3	308.7	22	22	308	344.5	395.1	424.6
Cariprazine	427.4	394.3	28	28	491	518.2	600.1	571.3
Dextromethorphan	271.4	280	20	20	370	376.3	394.9	411.5
Olanzapine	312.4	308.7	22	22	432	405.8	476	454.1
Tranylcypromine	133.2	134.4	10	10	116	113.5	218.3	248.2
Brexpiprazole	433.6	436.7	31	31	636	648.8	675.2	680.3
Mirtazapine	265.4	280	20	20	345	353.1	432.4	412.5
Venlafaxine	277.4	280	20	20	279	306.7	397.6	386.7
Gepirone	359.5	365.9	26	26	476	477.5	562.3	526.9
Vilazodone	441.5	464.8	33	33	729	729.5	745.1	737.2
Escitalopram	324.4	337.4	24	24	466	431.7	428.3	477.1
Paroxetine	329.4	337.4	24	24	402	433.7	451.7	492.5
Maprotiline	277.4	294.4	21	21	339	381.8	399.6	425.8
Levomilnacipran	246.4	251.1	18	18	295	266.5	393	345.6

**TABLE 15:** Comparison of physico-chemical properties with their predicted values.

Properties	EV		FP		MR		PO		MV	
Drug	Actual	Predicted	Actual	Predicted	Actual	Predicted	Actual	Predicted	Actual	Predicted
Trazodone	80.3	81.4	273.4	272.1	104.2	103.7	41.3	41.1	278.8	297.7
Bupropion	57.8	57	156.3	145	67.9	66.2	26.9	26.2	224.7	195.3
Vortioxetine	67.9	67.5	210.7	199.7	92.7	84.8	36.8	33.6	256.5	253.9
Sertraline	67	65.1	205.6	187.4	85.8	81.1	34	32.1	243.9	245.1
Aripiprazole	95.3	95	344.6	342.6	120.3	119	47.7	47.1	355	333.2
Desvenlafaxine	69.1	62.9	193.2	175.7	77.8	77.3	30.9	30.7	236.1	232.2
Citalopram	68.3	75.4	212.8	241	92.1	96.1	36.5	38.1	272.6	284
Fluoxetine	64.5	70	192.8	212.8	79.9	88.6	31.7	35.1	266.7	266.1
Cariprazine	89.3	87.9	316.7	305.9	117.2	111.3	46.4	44.1	344.5	317.5
Dextromethorphan	64.5	65.1	116.2	187.4	81.8	81.1	32.4	32.1	243.8	238.7
Olanzapine	74	70	241.7	212.8	92.2	88.6	36.5	35.1	236	255.1
Tranylcypromine	45.5	49	90.8	102.6	41.8	44	16.6	17.5	125	132
Brexpiprazole	99.1	98.7	362.1	362	126.3	122.8	50.1	48.6	348	339.2
Mirtazapine	68.8	65.1	215.3	187.4	80.7	81.1	32	32.1	216.6	237.4
Venlafaxine	68.3	65.1	194.2	187.4	82.6	81.1	32.8	32.1	261.7	247.6
Gepirone	84.5	81.4	293.8	272.1	99	103.7	39.3	41.1	314.7	296.6
Vilazodone	108.6	106.6	404.4	402.9	128.7	130.5	51	51.7	328.8	354.9
Escitalopram	68.3	75.4	212.8	241	92.1	96.1	36.5	38.1	272.6	284
Paroxetine	71.1	75.4	227	241	87.9	96.1	34.9	38.1	271.5	279.3
Maprotiline	65	67.5	187.7	199.7	87.8	84.8	34.8	33.6	256.7	255.1
Levomilnacipran	64.3	60.8	191.5	164.8	73.2	73.6	29	29.2	228.6	238.1

## 4 Validation

To ensure the robustness of the models, validation was carried out using both leave-one-out cross-validation $\left(Q_{\mathrm{LOO}}^{2}\right)$ and external dataset analysis.

### 4.1 Leave-one-out cross-validation

The $Q_{\mathrm{LOO}}^{2}$ metric evaluates the model’s performance by iteratively excluding one data point at a time and assessing how accurately the model predicts the omitted observation. It is considered a reliable indicator of model performance, especially for small datasets, as it helps determine the model’s ability to generalize to unseen data. An elevated $Q_{\mathrm{LOO}}^{2}$ value reflects strong predictive capability and confirms the overall reliability of the model. In addition to $Q_{\mathrm{LOO}}^{2}$ , the root mean square error of cross-validation (*RMSECV*), and absolute error (*MAE*) were also computed. The mathematical representation of the leave-one-out cross-validation coefficient $\left(Q_{\mathrm{LOO}}^{2}\right)$ is given by the following expression:

$$Q_{\mathrm{LOO}}^{2}=1-\frac{\sum_{j=1}^{n}{\left(y_{j}^{\mathrm{act}}-y_{j,LOO}^{\mathrm{pred}}\right)}^{2}}{\sum_{j=1}^{n}{\left(y_{j}^{\mathrm{act}}-{\bar{y}}_{j}^{\mathrm{act}}\right)}^{2}}$$

where $y_{j}^{\mathrm{act}}$ , $y_{j,LOO}^{\mathrm{pred}}$ , and ${\bar{y}}_{j}^{\mathrm{act}}$ represent the actual values in the dataset, the predicted values generated for each drug upon its exclusion from the training set with the model reconstructed using the remaining n−1 drugs, and the mean of actual values. $Q_{\mathrm{LOO}}^{2}$ , RMSECV , and MAE values are summarized in Table 16, with higher $Q_{\mathrm{LOO}}^{2}$ values reflecting stronger predictive capability.

**TABLE 16:** $Q_{\mathrm{LOO}}^{2}$ , RMSECV and MAE of leave-one-out cross-validation.

Properties	$Q_{\mathrm{LOO}}^{2}$	RMSECV	MAE
MW	0.9442	17.84	13.43
HAC	1	0	0
CO	0.9245	39.52	29.23
BP	0.8835	40.84	31.36
EV	0.838	5.78	4.06
FP	0.8558	28.88	21.73
MR	0.9288	5.32	4.16
PO	0.9303	2.08	1.63
MV	0.8368	20.82	16.44

### 4.2 External validation

External validation is a statistical measure used to assess the predictive power of a model using an independent external test set. It provides an estimate of the model’s performance on completely unseen data and further highlights its predictive capability. We have performed external validation using two drugs, namely, duloxetine (Mallinckrodt et al., 2006) and esketamine (Vasiliu, 2023), as the external test set. RMSE , and MAE were calculated and tabulated in Table 17, the external dataset is small making it difficult to draw reliable conclusions based solely on these metrics. Therefore, the residual values for the two drugs were also reported in Table 18 to further assess the predictive ability of the model.

**TABLE 17:** RMSE and MAE of external validation.

Properties	RMSE	MAE
MW	11.25	9.33
HAC	0	0
CO	31.51	27.25
BP	49.4	49.12
EV	4.7	4.66
FP	32.58	32.38
MR	4.44	3.36
PO	1.75	1.3
MV	5.15	5.11

**TABLE 18:** Residual values of the external drugs.

Properties	MW	HAC	CO	BP	EV	FP	MR	PO	MV
Duloxetine	3.039	0.000	−11.427	54.376	5.322	35.969	6.268	2.478	−4.466
Esketamine	15.614	0.000	43.075	43.873	3.990	28.793	−0.459	−0.131	−5.746

## 5 Discussion

To get insight from linear, quadratic, and logarithmic regression analyses, this study used a variety of characteristics, including correlation coefficients. These findings facilitate the development of mathematical models for predicting the physicochemical behavior of novel compounds. By correlating twelve degree-based indices and twelve neighborhood degree-based indices with the nine physicochemical properties using linear regression analysis, a total of 216 linear models were constructed and evaluated based on their correlation coefficients, from which nine best predictive linear models were identified. Similarly, quadratic and logarithmic regression analyses were performed, yielding 216 models each, and the corresponding correlation coefficients were systematically compared to determine the nine most predictive quadratic models and the nine most predictive logarithmic models. A comparative assessment of these twenty-seven shortlisted linear, quadratic, and logarithmic models ultimately led to the identification of the nine most effective models for predicting the physicochemical properties of the drugs. In the case of HAC, the linear regression model exhibited a perfect fit; therefore, the quadratic regression model was not considered further, as the simpler linear model was sufficient to describe the relationship. The nine most predictive models were developed using the degree-based indices $ReZ_{1}^{d}$ , $SO^{d}$ , $ABC^{d}$ , and ^*m*^ $M_{2}^{d}$ , among which $ReZ_{1}^{d}$ demonstrated the highest predictive capability by accurately modeling the maximum number of physicochemical properties. Additionally, validation studies were carried out to demonstrate the robustness of the model. Collectively, the findings of this study contribute novel predictive models for accurately estimating the physicochemical properties of major depressive disorder drug molecules.

## 6 Conclusion

This study utilized degree-based and neighborhood degree-based edge partitioning methods and SageMath 9.3 software to compute the relevant indices of major depressive disorder drugs. Using these indices, QSPR analyses were performed by constructing linear, quadratic, and logarithmic regression models and systematically comparing their predictive abilities. Upon comparison, linear regression models provide the most accurate prediction of the heavy atom count with an $r^{2}$ value of 1, and molecular weight, complexity, boiling point, enthalpy of vaporization, flash point, molar refractivity, polarizability, and molar volume are best predicted using quadratic models with $r^{2}$ values of 0.963, 0.9362, 0.9455, 0.9352, 0.9062, 0.9559, 0.9564, and 0.9101, respectively. Researchers may accurately anticipate the physicochemical characteristics of new medications for the treatment of major depressive disorder by using the most efficient QSPR models. This greatly cuts down on the time and expense needed for molecular preparation and clinical testing.

## Data Availability

The original contributions presented in the study are included in the article/supplementary material, further inquiries can be directed to the corresponding author.
